# An Observational Study of the Mental Health Burden in Frail and Elderly Patients

**DOI:** 10.1192/bjo.2022.230

**Published:** 2022-06-20

**Authors:** Yathorshan Shanthakumaran, Reshma Rasheed, Anjali Patel

**Affiliations:** 1 Rigg Milner Medical Centre, East Tilbury, United Kingdom; 2 University Hospitals Plymouth NHS Trust, Plymouth, United Kingdom; 3 New Vision Medical University, Tbilisi, Georgia

## Abstract

**Aims:**

Psychiatric illnesses are common among older adults and are associated with increased mortality and physical comorbidities. It is suggested that patients with frailty have a higher prevalence of depressive symptoms. (1) The eFI (electronic Frailty Index) is a tool used to assess the severity of frailty in elderly frail patients using a cumulative deficit model based on routine interactions with their GP.

**Methods:**

Patients were selected for annual frailty assessments by searching the electronic clinical system (SystmOne) using the eFI tool. Patients were assessed using the Comprehensive Geriatric Assessment (CGA) framework. In addition, all patients were screened for coexisting anxiety and depression using the Patient Health Questionnaire (PHQ-9) and Generalized Anxiety Disorder (GAD-7) questionnaire.

**Results:**

Of the 118 patients who ranged from mild to severe frailty, we found there was a positive correlation of the frailty severity eFI scores with increased rates of anxiety and depression evidenced by higher scores on the PHQ-9 and GAD-7 scoring tools. We found a positive correlation of the eFI with the PHQ-9 depression scores of (r = 0.819 p < 0.001). Within the same data set, we found correlation coefficients of eFI and anxiety GAD-7 scores (r = 0.651 p < 0.001). Increasing frailty was found to be associated with a higher rate of depression and anxiety.

**Conclusion:**

We found in this study higher (eFI) electronic frailty indices are associated with higher rates of anxiety and depression. We would recommend annual frailty assessments in patients with high electronic frailty indices and this should include screening for mental health deterioration. Early detection of deterioration will enable patient centered supportive measures and targeted treatment strategies. Health maintenance programs should ensure patient centered holistic assessment of both physical and mental health needs for early identification to avoid deterioration of both physical and mental health.

